# Association between glucose to lymphocyte ratio and prognosis in patients with solid tumors

**DOI:** 10.3389/fimmu.2024.1454393

**Published:** 2024-12-06

**Authors:** Rongqiang Liu, Yankun Shen, Jiahui Cui, Wangbin Ma, Jianguo Wang, Chen Chen, Weixing Wang

**Affiliations:** ^1^ Department of Hepatobiliary Surgery, Renmin Hospital of Wuhan University, Wuhan, Hubei, China; ^2^ Department of General Surgery, Zhongshan Hospital, Fudan University, Shanghai, China; ^3^ Department of Anesthesiology, Renmin Hospital of Wuhan University, Wuhan, Hubei, China

**Keywords:** glucose-to-lymphocyte ratio, tumor, prognosis, meta-analysis, survival

## Abstract

**Background:**

Glucose-to-lymphocyte ratio (GLR) plays an important role in the prognosis of various tumors. The aim of this study was to comprehensively evaluate the prognostic value of GLR in solid tumors through the meta-analysis.

**Methods:**

A comprehensive search of eligible studies was performed by scrutinizing the Pubmed, Embase and Web of science databases until May 30, 2024. The pooled hazard ratios (HRs) with 95% confidence intervals (CIs) were calculated to evaluate overall survival (OS), disease-free survival (DFS) and recurrence free survival (RFS).

**Results:**

A total of 22 studies from 14 articles involving 9472 patients were included in the study. The pooled analysis showed that cancer patients with high GLR was significantly associated with unfavorable OS (HR:1.48,95% CI:1.34-1.63) and DFS/RFS (HR:2.20,95% CI:1.66-2.92). Subgroup analysis further showed that high GLR had better predictive value in liver cancer (HR:2.66, 95%CI:1.80-3.93), breast cancer (HR:2.13, 95%CI:1.10-4.13) and pancreatic cancer (HR:1.92, 95%CI:1.30-2.84).

**Conclusions:**

GLR can be used as an effective prognostic marker in patients with solid tumors.

## Introduction

According to the World Health Organization, cancers have become the leading cause of human death (1). In China, colorectal, stomach, esophagus and liver cancers are also commonly diagnosed as the leading causes of cancer deaths (2). Despite tremendous progress in the prevention and treatment of cancer, the incidence and mortality of cancers continue to rise (3). Many cancer patients are diagnosed at advanced stages and miss the best time for treatment. Many effective prognostic markers have been used for cancers, but their clinical application is not satisfactory. Therefore, there is an urgent need to identify new and more effective prognostic markers for cancers.

Inflammatory, immune and nutritional status influence tumor biological behavior (4–6). Multiple immunoinflammatory or nutritional indicators have been used to assess the prognosis of patients with tumors (7–9). However, these indicators only reflect inflammation, immune and nutritional status, and do not embody the body’s metabolic status. Tumor prognosis is not only related to inflammation, immunity and nutritional status, but also closely associated with glucose metabolism (10). Therefore, a new prognostic marker that can indicate both inflammatory immune status and metabolic status is needed.

Glucose-to-lymphocyte ratio(GLR) composed of glucose and lymphocyte as a new prognostic marker, is believed that it can effectively reflect the body’s glucose metabolism and inflammatory immune status (11). GLR was found to play an important role in tumor prognosis. Navarro et al. suggested that preoperative GLR was an independent predictor of overall survival(OS) and disease-free survival (DFS) in gallbladder cancer (12). Yang et al. showed that GLR can independently predict the prognosis of patients with colorectal cancer (13). Yılmaz et al. found that GLR was a new prognostic biomarker in advanced hepatocellular carcinoma (14). Hannarici et al. revealed that GLR was found to be independently prognostic factor for both recurrence free survival (RFS) and OS in metastatic gastric cancer (15). Park et al. reported that elevated preoperative GLR was associated with aggressive tumor characteristics and was an independent predictor of poor OS in patients with pancreatic cancer (16). Ni et al. displayed that high GLR represented adverse prognosis in renal cell carcinoma patients (17). Yang et al. disclosed that GLR was independent prognostic factors for patients with non-small cell lung cancer (18). Zhang et al. proved that GLR had predictive value for the survival of patients with breast cancer (19). Liu et al. demonstrated that elevated preoperative GLR was remarkably associated with poorer prognosis in patients with esophageal cancer and melanoma (20). However, due to the limited number of patients in a single study, the reliability of the conclusions was insufficient. Therefore, we conducted a meta-analysis to synthesize and clarify the applicability of GLR as a prognostic marker in solid tumors.

## Material and methods

### Search strategy

Articles in electronic databases (Pubmed, Embase and Web of science) were retrieved until May 30, 2024. We used the following keywords: “glucose to lymphocyte ratio” OR “glucose-to-lymphocyte ratio”. Language restriction was not set. The titles, abstracts, full texts, and the possible references were screened to identify qualified studies.

### Inclusion and exclusion criteria

Three researchers independently conducted the literature search. The inclusion criteria were as follows: (1) investigated the relationship between GLR and survival outcomes in solid tumors. (2) provided sufficient data to calculate the hazard ratios (HRs) and 95% confidence intervals (CIs). The exclusion criteria were as follows: (1) insufficient data to calculate the 95% CIs and HRs; (2) abstracts, case reports, reviews and letters.

### Data extraction and quality assessment

The relevant information was extracted, such as the name of the first author, year of publication, country, cancer type, sample size, treatment methods, analysis types and survival outcomes. We assessed the quality of each study according to the Newcastle–Ottawa Quality Assessment Scale (NOS) (21). The multivariate analysis was preferred because it considered the confounding factors.

### Statistical analysis

All data analysis was performed using the STATA version 12.0 software (Stata Corporation, College Station, TX, USA). HRs and their corresponding 95% CIs were used to analyze the pooled data. A fixed effects model was used when I (2) was <50%. A random effects model was used when I (2) was >50% (22). The subgroup analysis was performed to further explore the prognostic value of GLR in solid tumors. Meta-regression was used to explore the sources of heterogeneity. Sensitivity analysis was used to test the stability of the results. Begg’s test, Egger’s test and trim-and-fill method were used to assess publication bias (23, 24). P<0.05 denoted statistical significance.

## Results

### Search results

**Figure 1:**
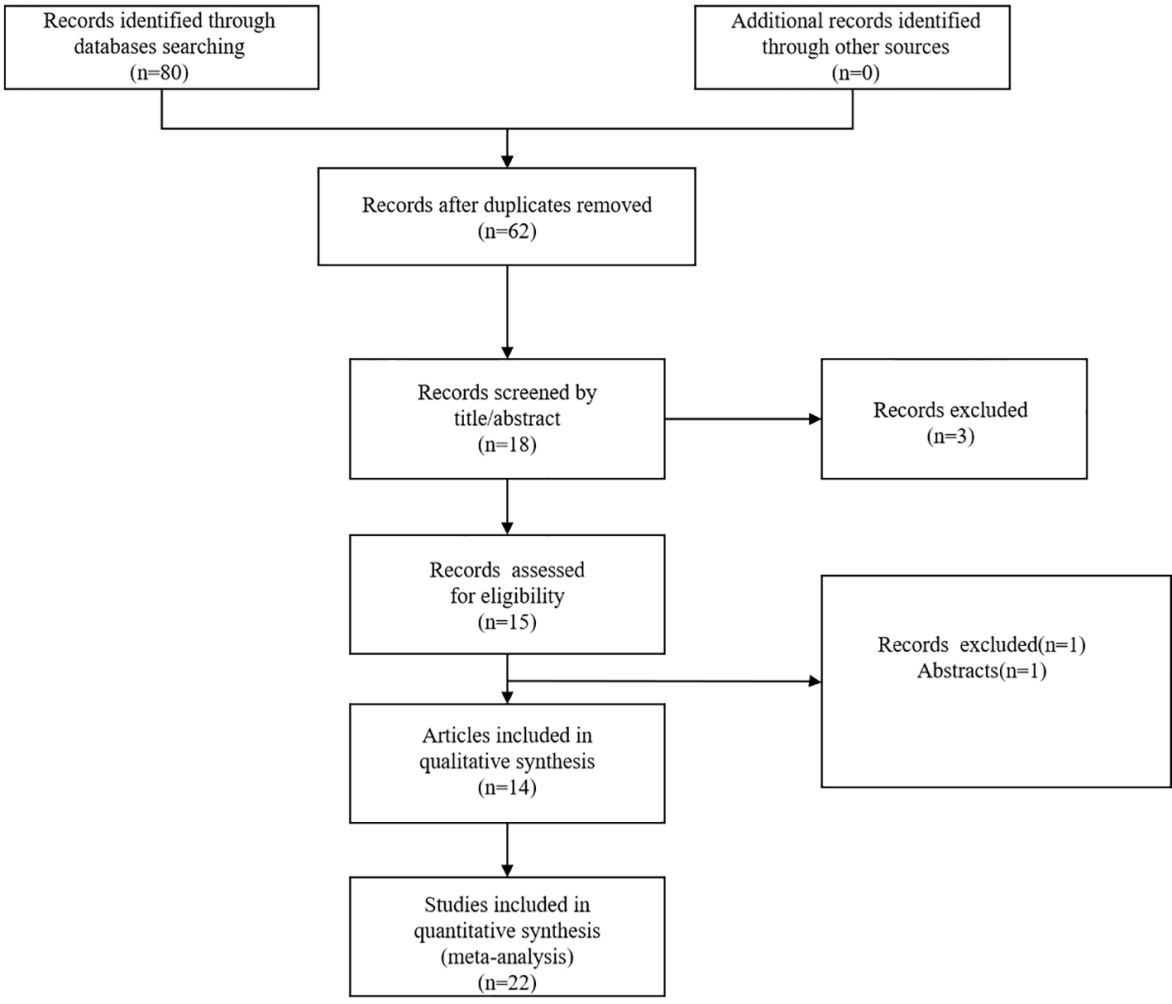
Flow diagram of the literature search.

Through a systematic literature search, we primarily identified a total of 80 articles. After removal of 62 duplicate publications, 18 articles remained. We further excluded 4 articles by browsing the titles and abstracts. Finally, we identified 22 studies from 14 articles published between 2019 and 2024 (9, 12–20, 25–28). The flow diagram of the literature search was shown in **Figure 1**.

### Study characteristics

The total number of patients in the included articles was 9472 (range: 110–1772 patients).18 studies were produced in China, 2 study were conducted in Korea and 2 study were from Turkey. 22 studies reported overall survival data, 1 study displayed disease-free survival data, and 3 studies covered recurrence free survival data. 10 different tumors were included, such as gastric cancer, gallbladder cancer, renal cancer, lung cancer, colorectal cancer, liver cancer, breast cancer, pancreatic cancer, esophageal cancer and melanoma. The NOS scores of the included studies ranged from 6 to 8 (mean: 6.5). The basic information was shown in (**Table 1**).

**Table 1:** Basic information of the included articles.

Study	Year	Country	Design	Cancer type	Sample size	Analysis type	Survival analysis	Treatment methods	NOS score
Hannarici	2023	Turkey	R	Gastric cancer	159	Multivariate analysis	OS,PFS	Non-surgery	7
Navarro	2019	Korea	R	Gallbladder Cancer	197	Multivariate analysis	OS,DFS	Surgery	7
Ni	2022	China	R	Renal cancer	420	Multivariate analysis	OS	Surgery	7
Song	2022	China	R	Lung cancer	1772	Multivariate analysis	OS	Non-surgery	8
Yang	2022	China	R	Lung cancer	862	Multivariate analysis	OS	Surgery	7
Yang	2022	China	R	Colorectal cancer	1448	Multivariate analysis	OS	Surgery	7
Yilmaz	2021	China	R	Liver cancer	150	Multivariate analysis	OS,PFS	Non-surgery	7
Yilmaz	2022	China	R	Breast cancer	110	Multivariate analysis	OS,PFS	Non-surgery	7
Zhang	2021A	China	R	Pancreatic cancer	130	Multivariate analysis	OS	Surgery	6
Zhang	2021B	China	R	Pancreatic cancer	129	Multivariate analysis	OS	Surgery	6
Zhong	2020	China	R	Pancreatic cancer	238	Multivariate analysis	OS	Non-surgery	6
Liu	2024A	China	R	Lung cancer	240	Multivariate analysis	OS	Surgery	6
Liu	2024B	China	R	Colorectal cancer	378	Multivariate analysis	OS	Surgery	6
Liu	2024C	China	R	Breast cancer	221	Multivariate analysis	OS	Surgery	6
Liu	2024D	China	R	Gastric cancer	335	Multivariate analysis	OS	Surgery	6
Liu	2024E	China	R	Liver cancer	270	Multivariate analysis	OS	Surgery	6
Liu	2024F	China	R	Esophageal Cancer	233	Multivariate analysis	OS	Surgery	6
Liu	2024H	China	R	Renal Cancer	295	Multivariate analysis	OS	Surgery	6
Liu	2024G	China	R	Melanoma	200	Multivariate analysis	OS	Surgery	6
Zhang	2023	China	R	Breast cancer	1125	Multivariate analysis	OS	Non-surgery	8
Aydin	2024	Turkey	R	Colorectal cancer	222	Multivariate analysis	OS	Surgery	6
Park	2024	Korea	R	Pancreatic cancer	338	Multivariate analysis	OS	Surgery	6

R, retrospective; OS, overall survival; DFS, disease-free survival; PFS, progression-free survival; NOS score, Newcastle-Ottawa Scale score.

### Association between high GLR and OS

**Figure 2:**
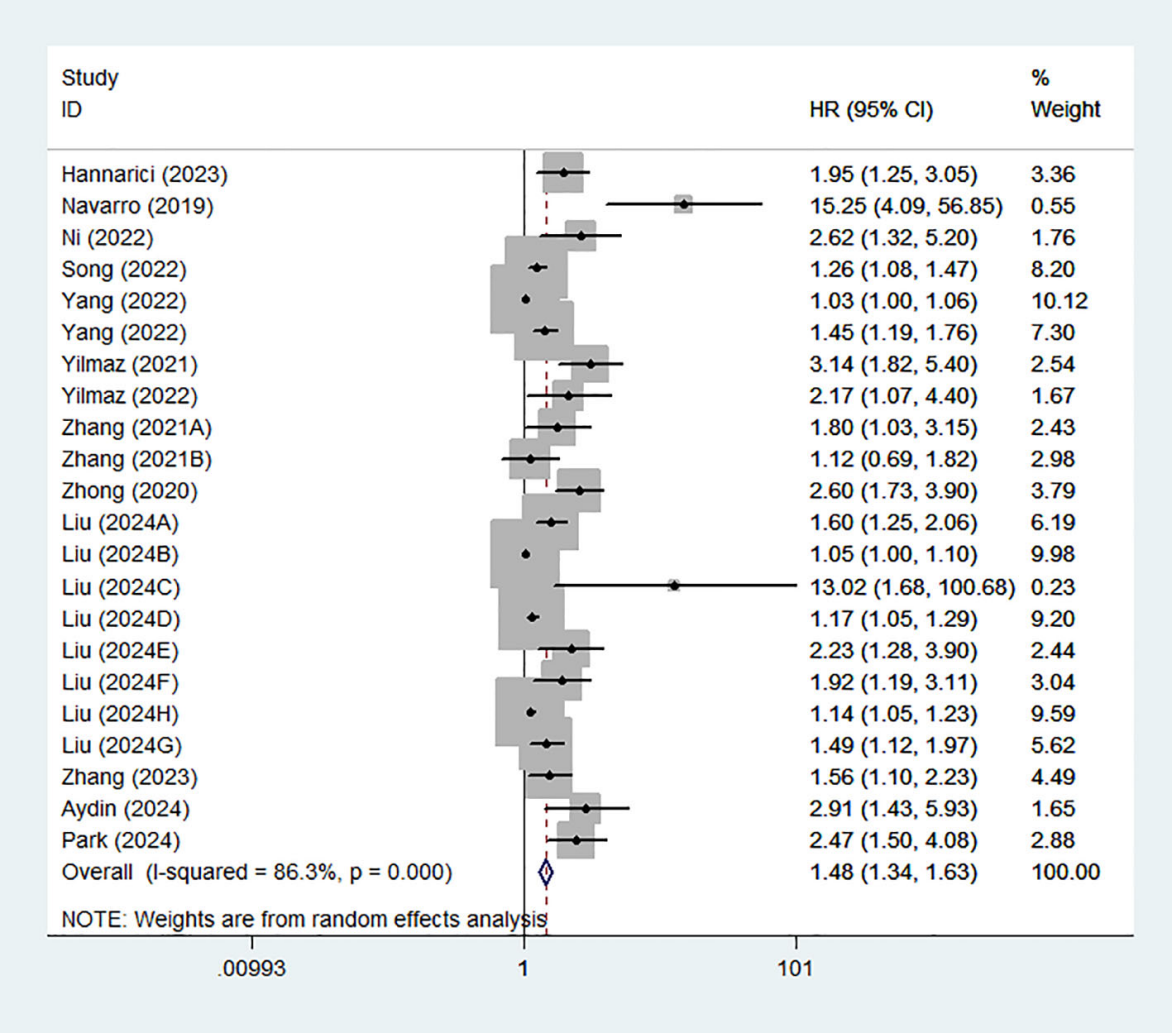
Forest plot assessing the relationship between GLR and OS.

22 studies from 14 articles explored the association between GLR and prognosis using OS. We used a random effects model to calculate the pooled HRs due to moderate heterogeneity (I^2^ = 86.3%). The results of the meta-analysis revealed that high GLR was significantly related to poor OS (HR:1.48,95% CI:1.34-1.63) (**Figure 2**).

### Subgroup analysis and meta-regression for OS

We further conducted subgroup analysis based on cancer type, sample size, treatment method and country. The results were shown in (**Table 2**). We found that high GLR was an unfavorable prognostic marker in liver cancer (HR:2.66, 95%CI:1.80-3.93), breast cancer (HR:2.13, 95%CI:1.10-4.13) and pancreatic cancer (HR:1.92, 95%CI:1.30-2.84). Moreover, we also found that high GLR was associated with poor OS for the China group (HR: 1.37; 95% CI:1.25–1.51) and Turkey group (HR:2.18; 95% CI: 1.50–3.19). Regardless of the surgical or non-surgical group, high GLR indicated adverse prognosis. Meta-regression showed that sample size was the main source of heterogeneity.

**Table 2:** Subgroup analysis for OS.

Factors	Studies	HR(95%)	P	Heterogeneity		Meta-regression		
				I^2^	P	Tau^2^	Adj R^2^(%)	P
Country						0.0889	20.13	0.93
China	18	1.37(1.25-1.51)	<0.001	84.9	<0.001			
Korea	2	5.525(0.94-32.43)	0.058	84.4	0.011			
Turkey	2	2.18(1.50-3.19)	<0.001	0	0.352			
Treatment method						0.1032	7.32	0.383
Non-surgery	6	1.93(1.40-2.66)	<0.001	76.6	0.001			
Surgery	16	1.35(1.22-1.49)	<0.001	85.1	<0.001			
Sample size						0.059	46.5	0.025
<250	12	2.08(1.64-2.63)	<0.001	62.2	0.002			
>250	10	1.21(1.11-1.32)	<0.001	83.9	<0.001			
Cancer type								
Gastric cancer	2	1.44(0.88-2.36)	0.148	79.1	0.029	0.092	17.01	0.139
Renal cancer	2	1.61(0.72-3.59)	0.248	82.2	0.018			
Lung cancer	3	1.24(0.98-1.57)	0.072	88.8	<0.001			
Colorectal cancer	3	1.21(0.98-2.01)	0.63	88.3	<0.001			
Liver cancer	2	2.66(1.80-3.93)	<0.001	0	0.393			
Breast cancer	4	2.13(1.10-4.13)	0.025	55.1	0.106			
Pancreatic cancer	4	1.92(1.30-2.84)	0.001	61.7	0.05			
Gallbladder Cancer	1	15.25(4.1-56.85)						
Esophageal Cancer	1	1.925(1.19-3.11)						
Melanoma	1	1.486(1.12-1.97)						

### Association between high GLR and DFS/PFS

**Figure 3:**
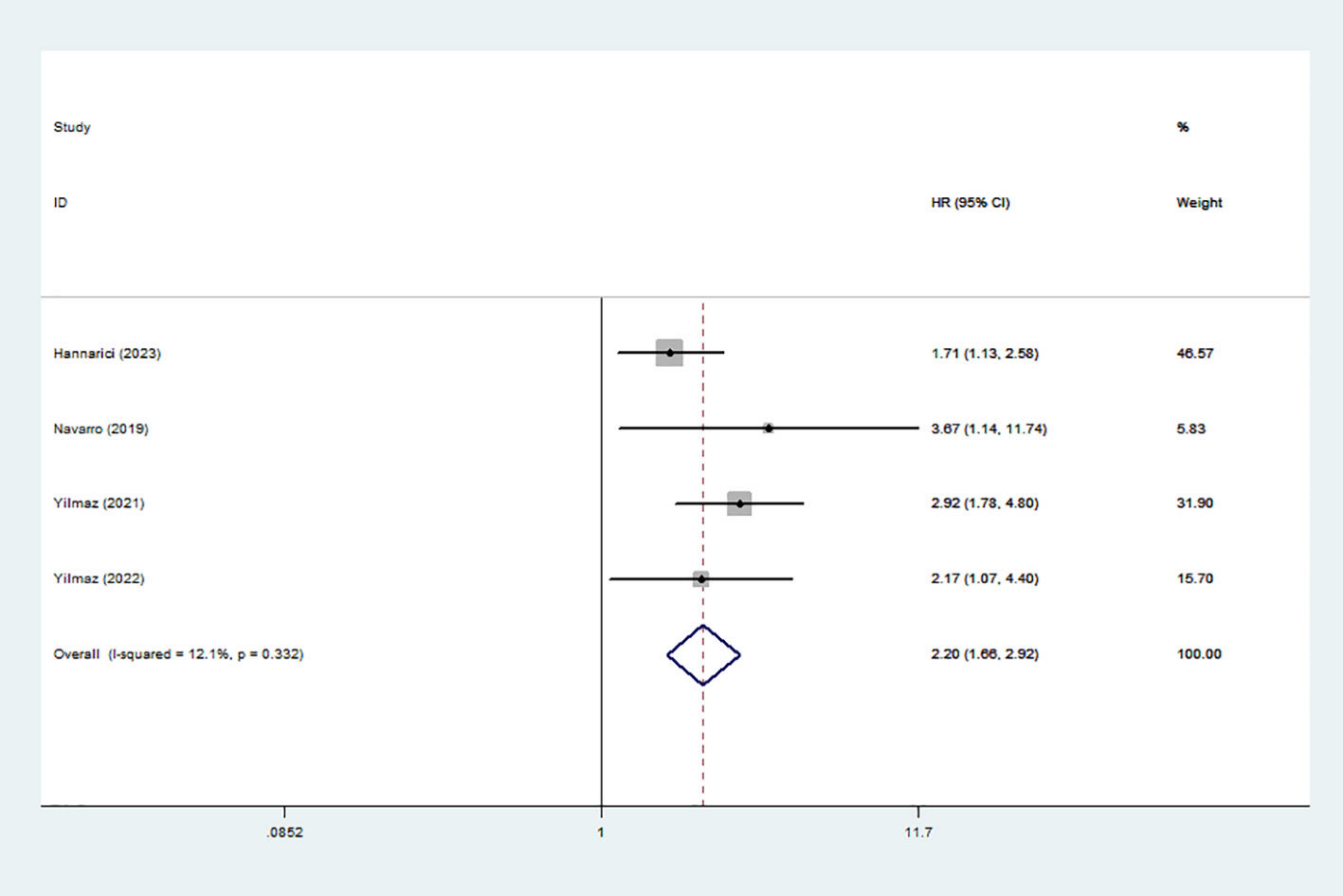
Forest plot accessing the relationship between GLR and DFS/PFS.

4 studies involving 616 patients documented the association between high GLR and prognosis using DFS/PFS. A fixed-effect model was used because of the obvious heterogeneity (I^2^ = 12.1%). The results showed that high GLR was correlated with adverse DFS/PFS (HR:2.20,95% CI:1.66-2.92) (**Figure 3**).

### Sensitivity analysis

**Figure 4:**
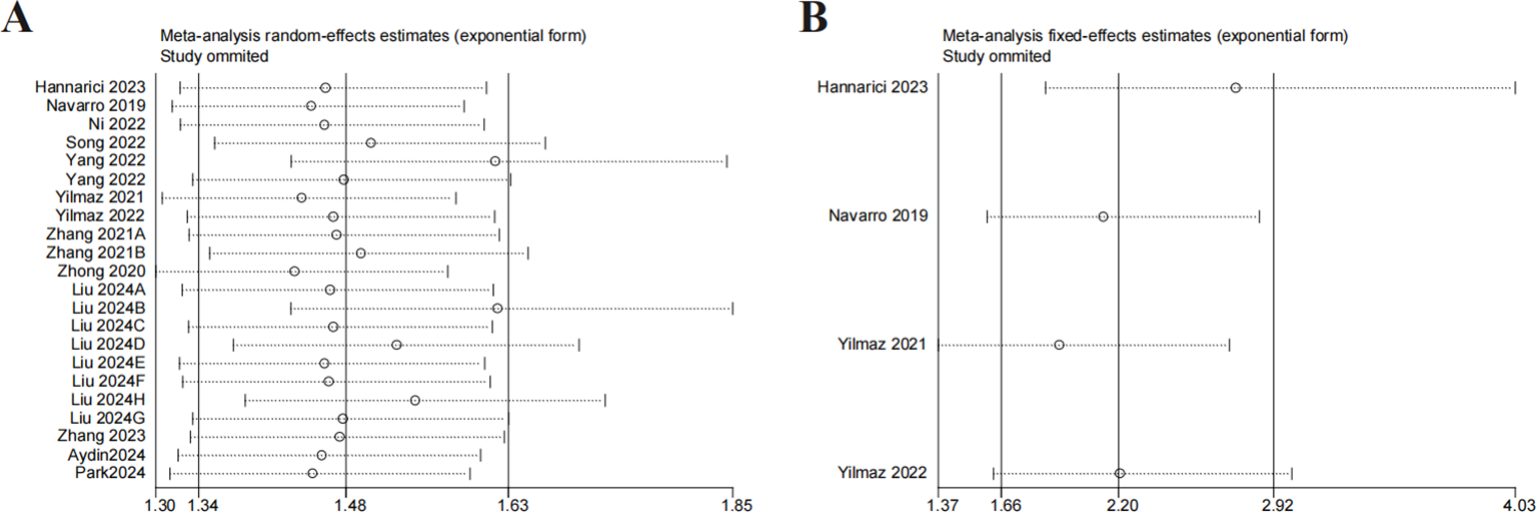
Sensitivity analysis. **(A)** sensitivity analysis for OS. **(B)** sensitivity analysis for DFS/PFS.

Sensitivity analysis was implemented by removing one study. The results were consistent with the comprehensive analysis, confirming that the outcomes of the combined OS and DFS/PFS were stable (**Figures 4A, B**).

### Publication bias

**Figure 5:**
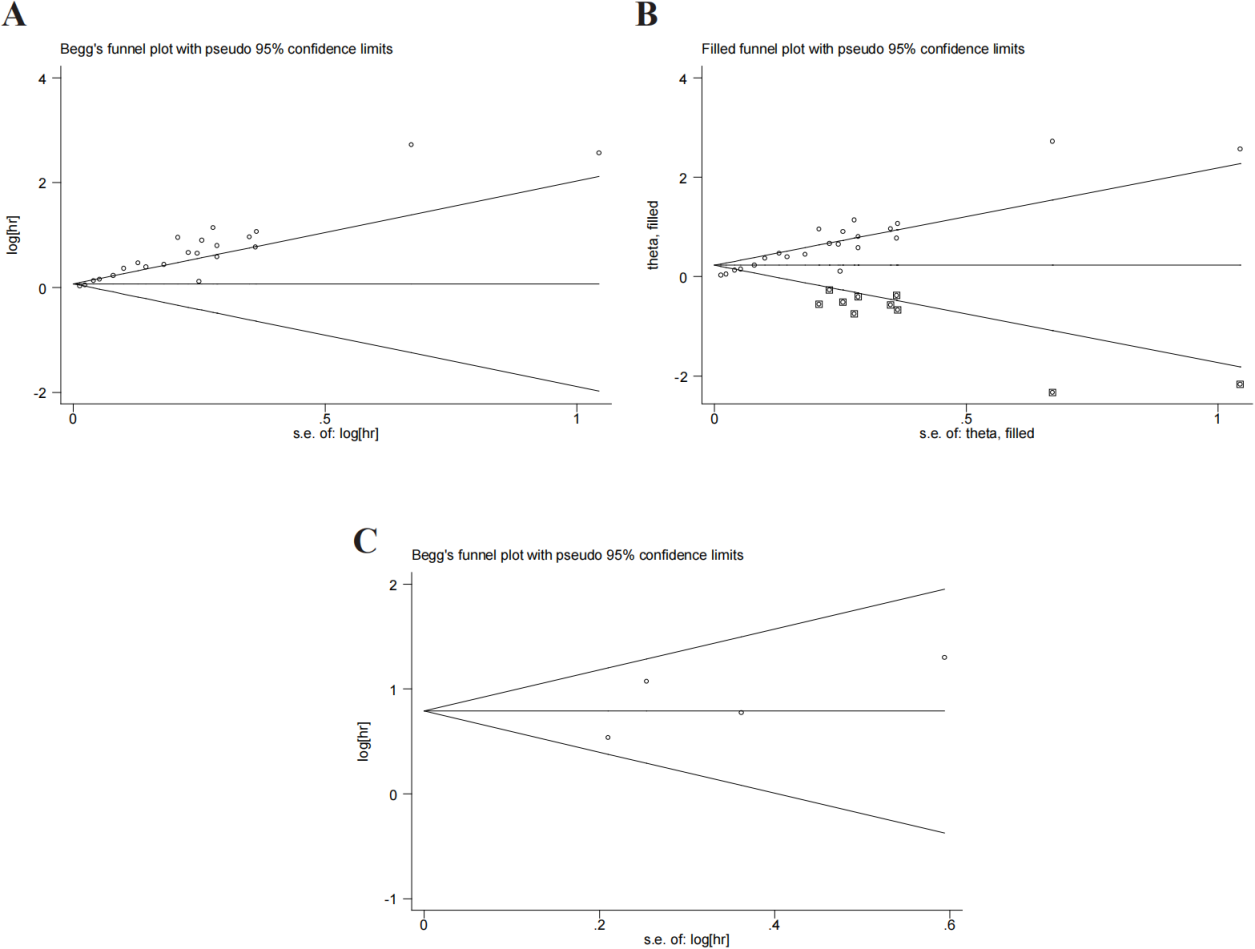
Publication bias. **(A)** publication bias for OS. **(B)** trim-and-fill method for OS. **(C)** publication bias for DFS/PFS.

Begg’s test and Egger’s test were used to evaluate the publication bias. P value of Begg’s test and Egger’s test for OS was 0.028 and 0.01 (**Figure 5A**), respectively. There was a degree of publication bias. However, we found that the comprehensive results were not affected through the trim-and-fill method (HR:1.258,95%CI:1.140-1.388) (**Figure 5B**). P values of Begg’s and Egger’s tests for DFS/PFS were 0.734 and 0.411, respectively (**Figure 5C**). P was more than 0.05 and no significant bias was observed.

## Discussion

To our knowledge, this study was the first meta-analysis to comprehensively assess the prognostic value of GLR in solid tumors. Our results suggested that high GLR was significantly associated with unfavorable OS and DFS/PFS in solid tumors. Subgroup analysis further showed that high GLR had better predictive value in liver cancer, breast cancer and pancreatic cancer.

GLR was firstly established as an effective prognostic indicator for gallbladder cancer (12). Subsequently, its prognostic value was confirmed in other cancers. In non-neoplastic diseases such as acute pancreatitis, myocardial infarction and acute respiratory distress syndrome, GLR also was shown to play an important role (29–31). Blood glucose was thought to be involved in the development of inflammation (32). The disturbance of glucose metabolism or hyperglycemia was found to promote the proliferation of tumor cells and increase the risk of death in patients (33). As one of immune cells, lymphocyte played a vital role in anti-tumor immune defense. Lymphocytopenia in tumor patients predicted poor prognosis (34). By combining blood glucose level and lymphocytes, GLR overcame the limitations of using blood glucose level or lymphocytes alone, and can more effectively reflect the metabolic, inflammatory and immune status of tumor patients.

GLR had significant advantages in predicting the prognosis of tumor patients by evaluating the metabolic, inflammatory and immune status of tumor patients. However, the specific mechanism that GLR affected the prognosis of tumor patients remained unclear. We tried to explain the phenomenon by the composition of GLR.

Blood glucose is an important component of human plasma, and is a good indicator of the body’s metabolic and endocrine functions. The survival of cancer cells is dependent on glucose. Hyperglycemia can promote the proliferation, invasion and migration of tumor cells, and enhance drug resistance of tumor cells (35). Hyperglycemia is conducive to the metabolic adaptation of tumor microenvironment and the maintenance of local immunosuppression (36). Hyperglycemia accelerates cancer progression by increasing reactive oxygen species levels (37). Elevated blood glucose levels produce many free radicals, leading to inflammation and metabolic disorders (38). Inflammation can accelerate cancer progression and lead to adverse survival (39). Evidence suggests that high blood glucose levels are associated with poor survival outcomes in a variety of tumors (40).

Lymphocyte as the important part of immune system plays an indispensable role in anti-tumor immune defense. Lymphocytes can inhibit tumor progression by directly inhibiting tumor cell proliferation (41). In addition, lymphocytes can activate cell-mediated immune responses and stimulate cytokines to promote tumor lysis (42). The data shows that T cells are more effective in suppressing anti-tumor immune response under hypoglycemic conditions (43). Accumulating evidences suggest that lymphocytes can reflect the nutritional status of patients (44). Studies have shown that high lymphocyte levels in the blood benefit the prognosis of patients with tumors, while lymphocytopenia may predict poorer survival outcomes (45, 46).

A high GLR indicated high glucose levels and a low lymphocyte count. The high GLR reflected more obvious the inflammation of tumor patients and the worse immune function of tumor patients. Therefore, it was not difficult to understand that high GLR was associated with a poor prognosis in patients with solid tumors.

There were some limitations in the study. Firstly, all articles had small sample sizes. Secondly, the included articles were retrospective studies. Thirdly, all studies included in the meta-analysis were conducted in Asia. More studies from other regions were warranted. Fourthly, publication bias for OS existed in the study. Finally, due to the lack of data, we were unable to assess the relationship between GLR and some pathological features.

Although there were some defects, the study also had some strengths. Firstly, we firstly demonstrated the prognostic value of GLR in solid tumors by meta-analysis. Secondly, the combined results were stable through sensitivity analysis. Thirdly, the trim-and-fill method found that the results for OS were unaffected by the publication bias. Finally, as a convenient serum marker, GLR can dynamically monitor the prognosis and therapeutic effect of patients with solid tumors.

In conclusions, we demonstrated that high GLR was associated with unfavorable survival outcome in solid tumors. GLR can serve as an effective prognostic indicator for patients with solid tumors, especially for liver, breast and pancreatic cancers. It can help doctors better identify high-risk patients so they can treat them more effectively. However, due to the shortcomings, more prospective studies were needed to confirm our findings.

## Data Availability

The original contributions presented in the study are included in the article/**Supplementary Material**. Further inquiries can be directed to the corresponding authors.
