# Supplementary figures and images for: Association between glucose to lymphocyte ratio and prognosis in patients with solid tumors
Source: Front Immunol. 2024 Dec 6;15:1454393. doi: 10.3389/fimmu.2024.1454393 (PMC11662397; doi:10.3389/fimmu.2024.1454393)

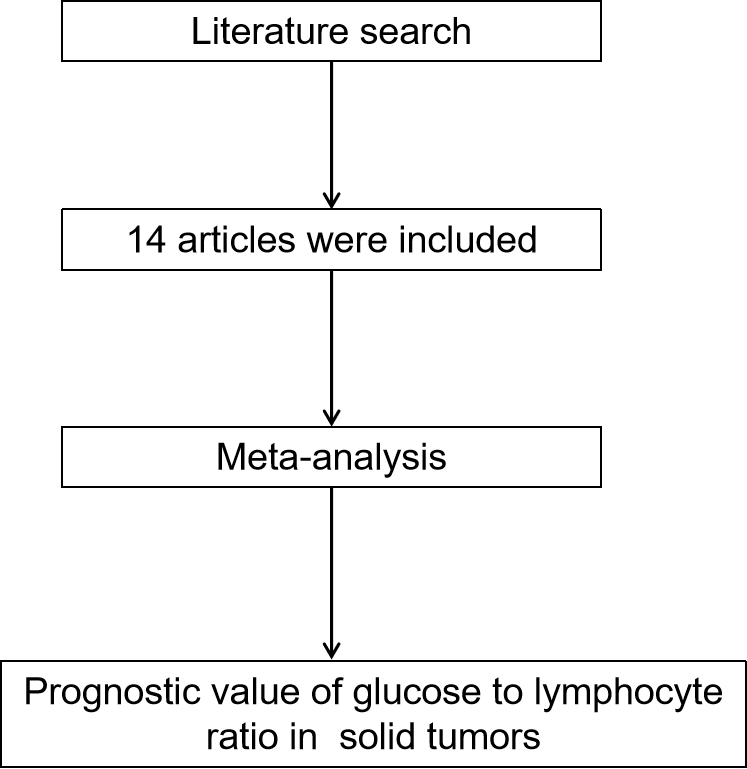

Supplement: Supplementary file 2 [file Image1.tif]
